# Region-specific RNA m^6^A methylation represents a new layer of control in the gene regulatory network in the mouse brain

**DOI:** 10.1098/rsob.170166

**Published:** 2017-09-20

**Authors:** Mengqi Chang, Hongyi Lv, Weilong Zhang, Chunhui Ma, Xue He, Shunli Zhao, Zhi-Wei Zhang, Yi-Xin Zeng, Shuhui Song, Yamei Niu, Wei-Min Tong

**Affiliations:** 1 Department of Pathology, Institute of Basic Medical Sciences and Neuroscience Center, Chinese Academy of Medical Sciences and Peking Union Medical College, Beijing 100005, People's Republic of China; 2 BIG Data Center, Beijing Institute of Genomics, Chinese Academy of Sciences, Beijing 100101, People's Republic of China; 3 University of Chinese Academy of Sciences, Beijing 100049, People's Republic of China; 4 State Key Lab of Molecular Oncology, National Cancer Center/Cancer Institute and Hospital, Chinese Academy of Medical Sciences and Peking Union Medical College, Beijing 100021, People's Republic of China

**Keywords:** N^6^-methyladenosine, RNA methylation, mouse cerebellum, mouse cerebral cortex, epitranscriptomic mark

## Abstract

N^6^-methyladenosine (m^6^A) is the most abundant epitranscriptomic mark found on mRNA and has important roles in various physiological processes. Despite the relatively high m^6^A levels in the brain, its potential functions in the brain remain largely unexplored. We performed a transcriptome-wide methylation analysis using the mouse brain to depict its region-specific methylation profile. RNA methylation levels in mouse cerebellum are generally higher than those in the cerebral cortex. Heterogeneity of RNA methylation exists across different brain regions and different types of neural cells including the mRNAs to be methylated, their methylation levels and methylation site selection. Common and region-specific methylation have different preferences for methylation site selection and thereby different impacts on their biological functions. In addition, high methylation levels of fragile X mental retardation protein (FMRP) target mRNAs suggest that m^6^A methylation is likely to be used for selective recognition of target mRNAs by FMRP in the synapse. Overall, we provide a region-specific map of RNA m^6^A methylation and characterize the distinct features of specific and common methylation in mouse cerebellum and cerebral cortex. Our results imply that RNA m^6^A methylation is a newly identified element in the region-specific gene regulatory network in the mouse brain.

## 1. Background

N^6^-methyladenosine (m^6^A) is a reversible mRNA epigenetic mark that has been shown to regulate RNA metabolism or structure by either facilitating or preventing methylation-dependent RNA–protein interaction [1–3]. An increasing body of evidence indicates that m^6^A methylation of nuclear mRNAs regulates pre-mRNA splicing [4,5], nuclear export [6] and pri-miRNA processing [7,8], while cytoplasmic methylated mRNAs are involved in translational control [9–15] and mRNA decay [16,17].

The importance of m^6^A in diverse biological processes has been investigated mainly through modulating the expression of m^6^A-related genes [18,19]. It was reported in yeast, *Arabidopsis*, zebrafish and *Drosophila* that m^6^A methyltransferases METTL3 and WTAP are essential for meiosis, development and viability [19–24]. In mammalian cells, both METTL3 and METTL14 are important for self-renewal and differentiation of mouse embryonic stem cells [24–29]. In addition, silencing of METTL3 led to circadian period elongation in mice [30]. RNA demethylase Alkbh5-deficient male mice showed defective spermatogenesis due to an imbalance in m^6^A levels [6]. Another RNA demethylase, FTO, is abundant in the brain and plays a regulatory role in adipogenesis, dopaminergic signalling and adult neurogenesis [31–35].

Like all physiological processes, neural activities rely on precise regulation of gene expression programmes at both genetic and epigenetic levels [36]. Epigenetic control of neurogenesis has been intensively investigated, with mechanisms including DNA methylation [37], histone modification [38], chromatin remodelling [39] and non-coding RNAs (lncRNAs) [40]. RNA modifications such as m^6^A, 5-methylcytidine (m^5^C) and N^1^-methyladenosine (m^1^A) have been proved to be essential regulatory elements in various biological processes [41–45]. Given a growing population of coding and lncRNAs identified in the brain, RNA modifications are thought to be important epitranscriptomic marks in the brain [46]. m^6^A was found to be dynamically regulated during brain development and correlated with memory formation [34,35,47,48]. However, despite the numerous experimental findings, the precise biological functions of m^6^A in the brain still await elucidation. Meanwhile, the brain is unique in its anatomical complexity and cellular heterogeneity [36,49,50], necessitating a detailed investigation into the regulatory role of m^6^A in the brain.

To explore the functional relevance of region-restricted m^6^A methylation in different brain regions, we performed the first transcriptome-wide m^6^A profiling analysis using adult mouse cerebellum and cerebral cortex.

## 2. Methods

### 2.1. Animals

All experiments were performed using wild-type, two-month-old C57/BL6 mice purchased from Vital River Co. (Beijing, China). All animal experiments and euthanasia were approved and performed in accordance with the guidelines of Animal Care and Use Committee of IBMS/PUMC.

### 2.2. RNA isolation

Mice were euthanized by cervical dislocation, and their cerebellum and cerebral cortex were dissected as described previously [51]. Mouse tissues were immediately snap-frozen into liquid nitrogen and then stored at −80°C until further use. Total RNA and poly(A) RNA were purified from frozen mouse cerebellum or cerebral cortex using TRI-Reagent (SIGMA) and FastTrack^®^ MAG mRNA Isolation Kits (LIFE). Poly(A) RNA purity was confirmed by using Agilent Bioanalyzer 2100. When needed, the poly(A) RNA will be purified again using the RiboMinus™ Human/Mouse Transcriptome Isolation Kit (LIFE).

RNA expression of m^6^A writers and erasers in the cerebellum and the cerebral cortex was measured by reverse transcription (TOYOBO) of total RNA and subsequent quantitative real-time PCR (TOYOBO). *Gapdh* was used as an internal control. The primers used in this study are listed in the electronic supplementary material, table S12.

### 2.3. Quantitative m^6^A level measurement using UHPLC-MS/MS

The global m^6^A methylation level of poly(A) RNA was measured using Agilent Technologies 6490 Triple Quadruple LC/MS as described before [6]. Briefly, RNA was digested with nuclease P1 (Sigma) at 37°C for 2 h, followed by treatment with calf intestine alkaline phosphatase (CIAP, Promega) at 37°C for 2 h. The solution was filtered and injected into the UHPLC-MS/MS system. The absolute m^6^A level in each sample was calculated by comparison with the standard curve obtained from pure nucleoside standards loaded simultaneously. The ratio of m^6^A to A was calculated to reflect the global methylation level.

### 2.4. Western blot analysis

Mouse cerebellum and cerebral cortex were dissected as described above and triturated in RIPA buffer supplemented with protease and phosphatase inhibitors; 60–80 µg of tissue lysates were subjected to SDS-PAGE and western blot analysis. Primary antibodies used in our analysis are as follows: anti-METTL3 (Abnova, H00056339-B01P), anti-METTL14 (Atlas Antibodies, HPA038002), anti-ALKBH5 (Sigma, HPA007196), anti-WTAP (Santa Cruz, sc-55438), anti-FTO (Abcam, ab92821) and beta-ACTIN (Santa Cruz, sc-47778). Secondary antibodies used are as follows: peroxidase-conjugated AffiniPure Goat Anti-Rabbit IgG (H + L) (XiYA Biology, Beijing, FZ-4201), peroxidase-conjugated AffiniPure Goat Anti-Mouse IgG (H + L) (XiYA Biology, Beijing, FZ-4202) and peroxidase-conjugated AffiniPure Rabbit Anti-Goat IgG (H + L) (XiYA Biology, Beijing, FZ-4203).

### 2.5. Immunohistochemical analysis

After being euthanized with tribromoethanol solution (4 mg g^−1^), mouse whole brain was dissected and post-fixed in 4% paraformaldehyde overnight at 4°C. Subsequently, the brain was dehydrated with ethanol, clarified with xylene and then embedded in paraffin. Sections 4 µm thick were used for immunostaining. Primary antibodies used in the analysis included: anti-METTL3 (Proteintech, 15073-1-AP), anti-METTL14 (Atlas Antibodies, HPA038002), anti-WTAP (Proteintech, 60188-1-IG), anti-ALKBH5 (Sigma, HPA007196) and anti-FTO (Abcam, AB92821). Secondary antibodies included: ImmPRESS Anti-Mouse Ig (peroxidase) Kit (Vector, MP-7402) and ImmPRESS Anti-Rabbit Ig (peroxidase) Kit (Vector, MP-7401). Staining of brain sections was visualized using a Panoramic MIDI II digital slide scanner (3D HISTECH).

### 2.6. m^6^A-immunoprecipitation

For each m^6^A-immunoprecipitation (IP) reaction, cerebellar RNA was pooled from three male and three female mice, while cortical RNA was pooled from one male and one female mouse. Poly(A) RNA fragmentation was performed using RNA Fragmentation Reagents (Ambion) as instructed by the manufacturer. Five micrograms of fragmented poly(A) RNA was incubated with 12.5 µg of anti-m^6^A antibody (Synaptic System) at 4°C for 2 h, followed by addition of protein A-Sepharose 4B (Sigma). After overnight incubation at 4°C, beads were washed five times with IPP buffer (150 mM NaCl, 0.1% NP-40, 10 mM Tris–HCl, pH 7.4). Immunoprecipitated RNA was recovered by competitive elution with m^6^A and subsequent ethanol precipitation. Input and immunoprecipitated RNA products were used for cDNA library construction using the TruSeq RNA Sample Prep Kit protocol (Illumina), and were subjected to a 2 × 100 paired-end sequencing run using the Illumina Hiseq 3000 system. Two sets of biological replicates were performed to obtain reproducible results.

### 2.7. Data processing and reads mapping

For each sample, single-end reads (R2) were used for bioinformatic analysis. The quality control of raw data was evaluated using the FastQC software (v. 0.10.1). Sequencing data were preprocessed with in-house Perl scripts following three criteria: (i) the adaptor sequence was removed by finding the sequence GATCGGAAGA with at most two mismatched bases; (ii) the read bases with low quality score (less than 20) were trimmed off from the 3′-end; (iii) the reads longer than 20 nt and with high quality score (more than 70% bases with quality score greater than 25) were retained. The filtered reads longer than 50 nt were mapped against the mouse genome (mm10), allowing up to two mismatches using the TopHat software (v. 2.0.13) [52,53]. Only uniquely mapped reads were kept for the downstream analysis.

### 2.8. RNA expression analysis

Fragments per kilobase of transcript per million mapped read (FPKM) values for each gene in the cerebellum and the cortex were calculated by the Cufflinks toolkit (v. 2.0.2) [54]. Two biological replicates of each sample were combined for calculating FPKM. Transcripts with FPKM value larger than 0.2 were considered as stably expressed transcripts [55].

### 2.9. M^6^A peak calling and motif analysis

M^6^A peak calling was performed using the exomePeak software (v. 2.7.0) with a cut-off of the false discovery rate (FDR) less than 5% [56,57]. Only the m^6^A peaks having an overlap (greater than 50% in length) between the two replicates are considered as concordant m^6^A peaks and are used for the subsequent analysis intersectBed from BEDTools (v. 2.26.0) [58]. The common and specific m^6^A peaks were defined using the following criteria: (i) the common m^6^A peaks appear in the two biological replicates in both the cerebellum and the cortex; (ii) the specific m^6^A peaks appear in the two biological replicates of the cerebellum or the cortex, but not in any replicate of the other brain region. Commonly methylated mRNAs (CMRs) were defined as mRNAs containing common m^6^A peaks, while specifically methylated mRNAs (SMRs) were defined as mRNAs containing specific m^6^A peaks. Consensus sequence motifs enriched in m^6^A peaks were identified by Homer [59]. To verify the results obtained using the exomePeak software, peak calling analysis was also performed in parallel using the MACS software with the default parameters [60,61].

### 2.10. Characterization of m^6^A peak distribution patterns

The distribution of m^6^A peaks were characterized as previously described with minor modification [27,62]. A mouse reference transcriptome was generated first by using the longest transcript for each gene to characterize the distribution patterns of m^6^A peaks. For each transcript of protein-coding genes, 100 bins of equal length were split for the 5′UTR, coding sequence (CDS) and 3′UTR, respectively. For long non-coding RNA (lncRNA), the entire length of lncRNA was split into 100 bins with equal length. The percentage showing the number of m^6^A peaks in each bin is calculated to represent the occupancy of m^6^A peaks along the whole transcript.

To determine the distribution of m^6^A peaks, we divided the longest transcript of protein-coding genes into five regions, namely the 5′UTR region, start codon region, CDS region, stop codon region and 3′UTR region. For those transcripts with a 5′UTR or 3′UTR longer than 100 nt and a CDS longer than 200 nt, a 200 nt region centred on start codons or stop codons was defined as start codon region or stop codon region. For the transcripts whose 5′UTR or 3′UTR was shorter than 100 nt, the corresponding UTR regions were classified as start codon regions or stop codon regions. If the entire length of the CDS was less than 200 nt, the first half of the CDS was classified as the start codon region and the remaining sequence as the stop codon region. The m^6^A peaks of both the cerebellum and the cortex were mapped to each region using intersectBEDTools. If one m^6^A peak was mapped to more than one region, the priority of classification was set in the following order: (1) stop codon region, (2) start codon region, (3) CDS region, (4) 3′UTR region and (5) 5′UTR region. The number of m^6^A peaks in each region was calculated using the method described above.

### 2.11. Gene Ontology analysis

The DAVID tool with default parameters was used for Gene Ontology (GO) analysis [63]. Enriched GO terms shown in the main figures were manually curated, and a list of all selected terms of the biological process, cellular components and molecular functions category are provided in the electronic supplementary material tables.

### 2.12. Statistical analysis

Two-tailed Student's *t*-test was performed for statistical analysis of results in LC/MS and real-time qPCR. The Wilcoxon test was used for all bioinformatic analysis.

## 3. Results

### 3.1. Ubiquitous expression of m^6^A writers and erasers in mouse cerebellum and cerebral cortex

To obtain a complete view of m^6^A RNA methylation profiles from different tissues, we quantified the m^6^A content in various adult mouse tissues using an UHPLC-MS/MS assay. Although m^6^A was present in all tested tissues, RNA methylation levels were the highest in the brain (electronic supplementary material, figure S1*a*). In agreement with the structural complexity in the brain, RNA methylation levels in the brainstem, olfactory bulb, cortex, cerebellum and thalamus varied from each other. In this study, we chose mouse cerebellum and cerebral cortex for all subsequent analysis.

**Figure 1.**
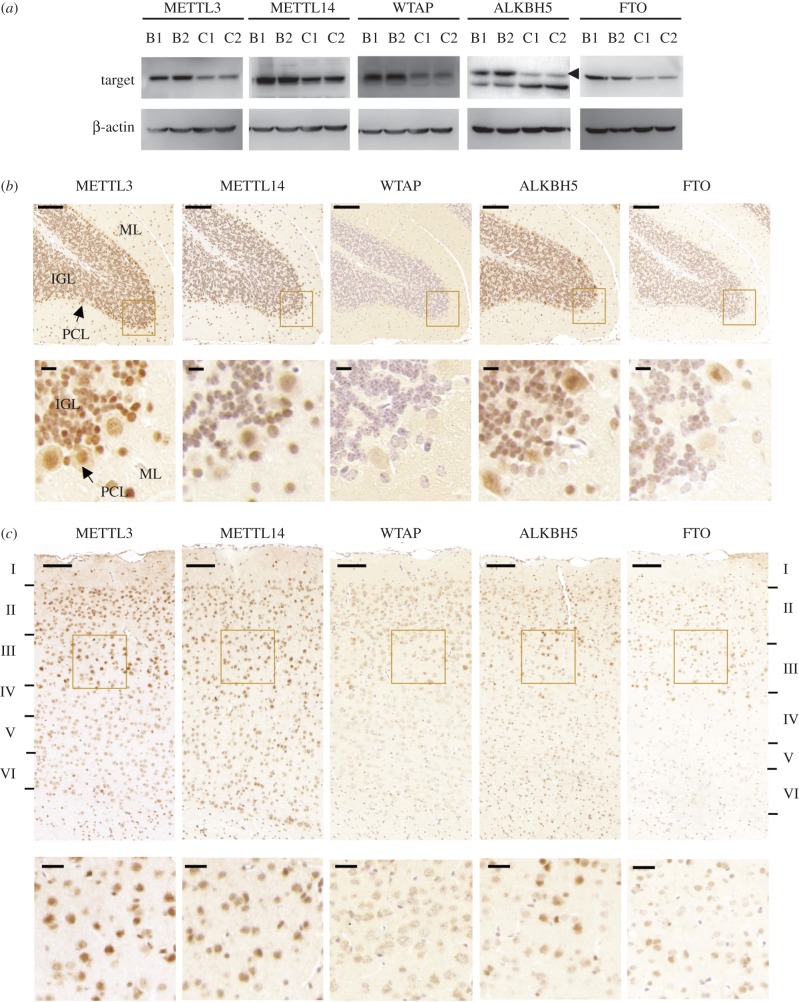
Protein expression of m^6^A writer and eraser genes in adult mice cerebellum and cerebral cortex. (*a*) Western blot analysis of METTL3, METTL14, WTAP, ALKBH5 and FTO in adult mouse cerebellum (B1, B2) and cerebral cortex (C1, C2). Beta-actin was used here as an internal control. Experiments were performed in biological triplicates using six mice in total. Representative results are shown here. (*b,c*) Representative images of immunostaining of METTL3, METTL14, WTAP, ALKBH5 and FTO using paraffin sections of adult mice cerebellum (*b*) and cerebral cortex (*c*). ML, molecular layer; IGL, inner granule cell layer; PCL, Purkinje cell layer. Scale bars represent 200 µm. The laminar structure of the cortex was labelled from layer I to layer VI. Experiments were performed in biological triplicates using three male and three female mice in total and representative data are given here. Enlarged images in the square area are shown in the lower panels and scale bars represent 10 µm in (*b*) and 50 µm in (*c*).

To gain a better understanding of the regional regulation of m^6^A methylation, we examined the expression profiles of five methyltransferases and demethylases: METTL3, METTL14, WTAP, ALKBH5 and FTO (figure 1*a* and electronic supplementary material, figure S1*b*). Notably, both protein and RNA expression levels of methyltransferases and demethylases appeared to be higher in the cerebellum than those in the cerebral cortex. To examine the *in situ* protein expression, we performed immunohistochemical staining. As shown in figure 1*b*, all five proteins were readily detected in the molecular layer (ML), Purkinje cells layer (PCL) and internal granular layer (IGL) of the mouse cerebellum, albeit with varying staining intensities. Similarly, all five proteins were ubiquitously expressed in the cerebral cortex with different levels among each layer (figure 1*c*). The ubiquitous expression of these proteins indicated that m^6^A methylation plays a role in various types of neural cells.

### 3.2. Distinct m^6^A methylation patterns between mouse cerebellar and cerebral cortical RNAs

**Figure 2.**
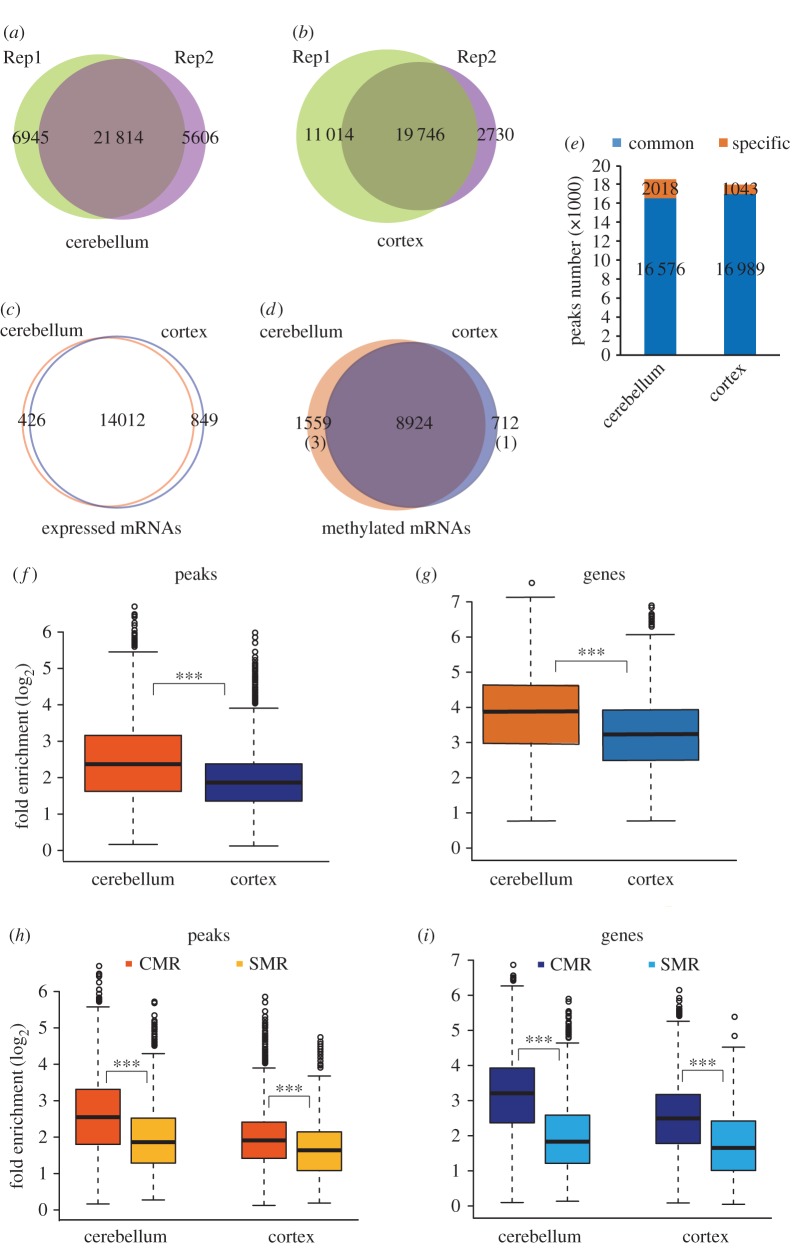
Region-specific m^6^A methylation in the mouse cerebellum and cerebral cortex. (*a, b*) Venn diagrams showing the numbers of overlapping m^6^A transcripts in the two biological replicates of m^6^A-IP in the cerebellum (*a*) and the cerebral cortex (*b*). (*c*) Venn diagram showing the numbers of genes commonly or specifically expressed in the cerebellum or the cerebral cortex. (*d*) Venn diagram showing the numbers of CMRs and SMRs. Numbers of specifically expressed genes among SMRs are shown in parentheses. (*e*) Column chart showing the numbers of common and specific m^6^A peaks in mouse cerebellum and cerebral cortex. The blue bars indicate common peaks, while the orange bars indicate specific peaks. (*f, g*) Box plots showing the methylation levels of cerebellar RNAs and cortical RNAs by comparing the median fold enrichment at peak levels (*f*) and gene levels (*g*). (*h, i*) Box plots showing the methylation levels of CMRs and SMRs by comparing the fold enrichment at the peak level (*h*) or gene level (*i*). Wilcoxon test was performed for statistical analysis. ***p value < 2.2 × 10^−16^.

To assess whether m^6^A methylation represents an important epigenetic mark in the mouse cerebellum and cerebral cortex, we conducted a transcriptome-wide m^6^A-seq analysis separately. Concordant m^6^A peaks from the two biological replicates were used for subsequent bioinformatic analysis (figure 2*a,b*, electronic supplementary material, table S1).

Although similar numbers of mRNAs were expressed (figure 2*c*; electronic supplementary material, table S2), the absolute numbers of methylated mRNAs and methylation sites were higher in the cerebellum than in the cortex (electronic supplementary material, table S3; figure 2*d,e*). Comparison between the two brain regions revealed the existence of both CMRs and SMRs (figure 2*d*, electronic supplementary material, figure S3 and table S3). In spite of the lower numbers of specifically expressed mRNAs in the cerebellum (426) than in the cortex (849) (figure 2*c*, electronic supplementary material, table S2), the number of SMRs was much higher in the cerebellum (1559) than in the cortex (712) (figure 2*d*, electronic supplementary material, table S3). Consistently, cerebellar mRNAs had more specific methylation sites than cortical mRNAs (figure 2*e*). Among these SMRs, only a few were specifically expressed (three specifically expressed genes in the cerebellum and one in the cortex), implying that RNA methylation constitutes an additional layer of regulation in a region-specific manner. Furthermore, the methylation levels between the cerebellum and the cortex were compared. As shown in figure 2*f,g*, the cerebellar mRNAs had significantly higher methylation levels than cortical mRNAs at both peak levels and gene levels (electronic supplementary material, figure S2*a* & S2*b*). Compared with the SMRs, the CMRs contained more peaks with higher fold enrichment (figure 2*h*, electronic supplementary material, figure S2*c,* S2*d*, and table S3), resulting in a more significant difference in methylation levels between CMRs and SMRs (figure 2*i*, electronic supplementary material, figure S2*e*, S2*f*). Considering that the largely different numbers of CMRs and SMRs may generate a difference when comparing their methylation levels, we randomly extracted the same numbers of peaks or RNAs from CMRs for a total of 10 times, which also showed significantly higher fold enrichment than those of SMRs (electronic supplementary material, figure S2*g,* S2*h*).

Considering the biological importance of lncRNAs in the brain, we also analysed the methylation profiles of lncRNAs in the two brain regions. The proportion of methylated lncRNAs, as well as the average number of m^6^A peaks per transcript, was much lower than those of methylated mRNAs (electronic supplementary material, table S2 and S3). As shown in the electronic supplementary material, figure S4*a* and S4*b*, in spite of comparable numbers of lncRNAs detected in the two brain regions, the cerebellum contained more methylated lncRNAs than the cortex samples. However, the proportion of specifically methylated lncRNAs was higher than those of mRNAs, suggesting that to exert their functions, lncRNAs exhibit a higher requirement for brain region-specific methylation (electronic supplementary material, figure S4*b*). The common and specific m^6^A methylation of lncRNAs in both regions were visualized from several randomly selected lncRNAs including *Malat1, Dlx4os* and *Miat* (electronic supplementary material, figure S4*c*).

We next evaluated how RNA methylation had an impact on RNA expression by using the input sample from the same experiment. We found that the median expression levels of methylated mRNAs were much higher than those of non-methylated mRNAs (electronic supplementary material, figure S5*a*). However, among all the methylated mRNAs, we observed a negative correlation between RNA abundance and RNA methylation levels (electronic supplementary material, figure S5*b,*S5*c*). Interestingly, the RNA abundance of the CMRs was significantly higher than that of SMRs in both the cerebellum and the cerebral cortex (electronic supplementary material, figure S5*d*).

Taken together, this comparative analysis revealed that the methylation levels of cerebellar RNAs were higher than those of cortical RNAs. CMRs and SMRs differed from each other in their methylation levels and overall RNA abundance, suggesting that common and specific methylation regulate gene expression in different ways.

### 3.3. Different distribution patterns between common and specific m^6^A peaks

**Figure 3.**
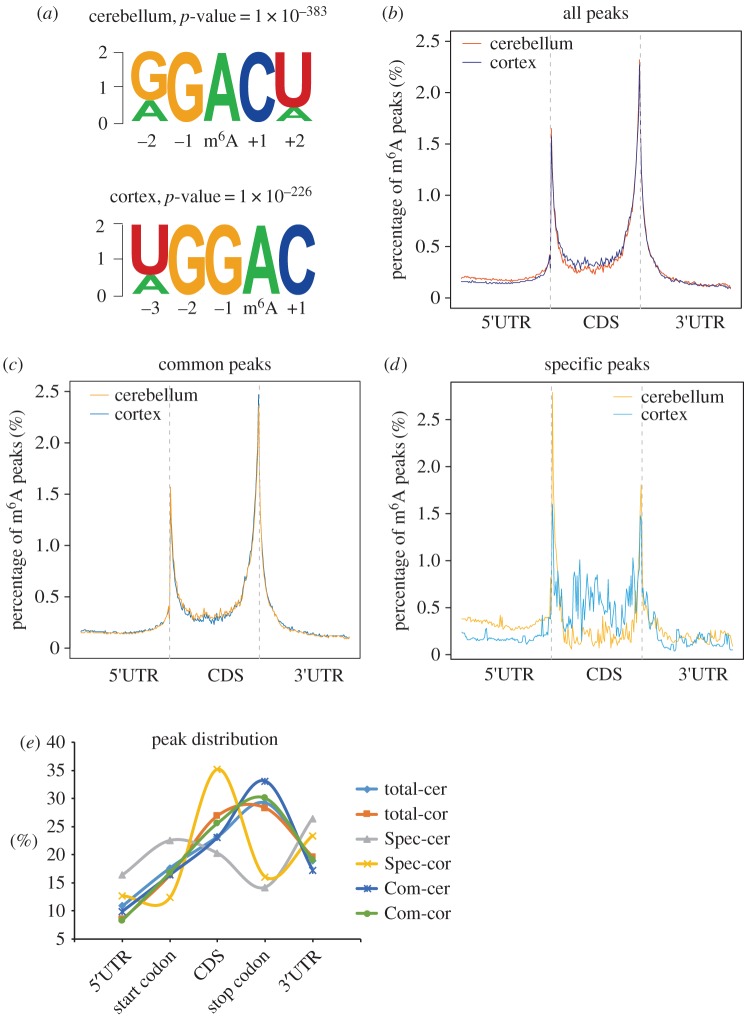
Distribution patterns of m^6^A peaks in mouse cerebellar and cerebral cortical mRNAs. (*a*) Sequence logo representing the deduced consensus motif through clustering of all enriched m^6^A peaks in the cerebellum and the cerebral cortex. (*b–d*) Enrichment of all m^6^A peaks (*b*), common m^6^A peaks (*c*) and specific m^6^A peaks (*d*) along the whole mRNA transcripts. (*e*) Statistics of numbers of m^6^A peaks enriched in different regions along the mRNA transcripts. Total m^6^A peaks in the cerebellum (total-cer) and cortex (total-cor); specific m^6^A peaks in the cerebellum (Spec-cer) and cortex (Spec-cor); common m^6^A peaks in the cerebellum (Com-cer) and cortex (Com-cor) were included and compared.

In most cases, m^6^A methylation does not occur randomly along the transcript but at specific consensus sequences and enrichment sites. To test whether this characteristic also exists in cerebellar and cortical RNAs, we performed a motif-searching analysis with all m^6^A peaks and found that GGACU/UGGAC were the most conserved consensus motifs in both the cerebellum and the cortex (figure 3*a*, electronic supplementary material, figure S6*a–d*).

We next analysed the distribution patterns of m^6^A peaks along the transcript. In addition to previously reported enrichment sites in the stop codon, a pronounced enrichment was also found surrounding the start codons in both the cerebellum and the cortex (figure 3*b*, electronic supplementary material, figure S6*e*). Common m^6^A peaks exhibited similar distribution patterns because they accounted for more than 75% of m^6^A peaks (figure 3*c*; electronic supplementary material, table S4 and S5). In those cerebellum-specific methylated transcripts, more m^6^A peaks were detected in the vicinity of start codons rather than stop codons. By contrast, cortex-specific m^6^A peaks were located near start codons and stop codons, as well as in the CDS (figure 3*d*, electronic supplementary material, table S6 and S7). To investigate the distribution patterns of common and specific peaks in detail, we analysed the peak numbers enriched in each gene region between the cerebellum and the cortex (figure 3*e*). When taking all the methylation sites into consideration, m^6^A sites were most abundant near stop codons and the 3′UTR, which was similar to previous reports. Notably, common and specific m^6^A peaks exhibited very different distribution patterns. The common m^6^A peaks were predominantly distributed near stop codons and the 3′UTR. By contrast, cerebellum-specific peaks were mainly distributed in start codons and 3′UTR regions, whereas cortex-specific peaks were mainly distributed in the CDS region.

In addition to m^6^A, N^6^, 2′-O-dimethyladenosine (m^6^Am) is another RNA modification found at the first nucleotide downstream from the m^7^G cap in the 5′UTR of mRNAs [64]. As the anti-m^6^A antibody has a cross-reactivity with m^6^Am, the length of the 5′UTR was calculated to test if the m^6^A observed at the start codon was due to the presence of m^6^Am. Among CMRs and cortical SMRs that contained m^6^A peaks surrounding start codons, more than 70% of them had a 5′UTR longer than 100 nt, while 60% of cerebellar SMRs had a 5′UTR longer than 100 nt (electronic supplementary material, figure S6*f*). Furthermore, m^6^Am was detected in only 30% of mRNA caps with a ratio of m^6^A to m^6^Am of approximately 30 [65]. Thus, the methylation sites surrounding start codons in our study are most probably m^6^A, rather than m^6^Am. However, for those methylated transcripts with a 5′UTR shorter than 100 nt, single-nucleotide resolution analysis should be performed to distinguish between m^6^A and m^6^Am [66].

### 3.4. Higher m^6^A methylation levels in neurons than in glial cells among the cell type-enriched RNAs

The mammalian brain is the most sophisticated organ ever studied, exhibiting considerable structural complexity and cellular diversity. With the discovery of additional epigenetic marks on mRNA, characterization of their functions in different cell types is essential for a more detailed understanding of the intricate mechanisms governing the brain functions. However, the current m^6^A-seq analysis is unable to discriminate RNA methylation among different cell types; we therefore analysed the cell type-enriched genes identified previously to have a first overview of the methylation profiles in different types of neural cells [67,68].

**Figure 4.**
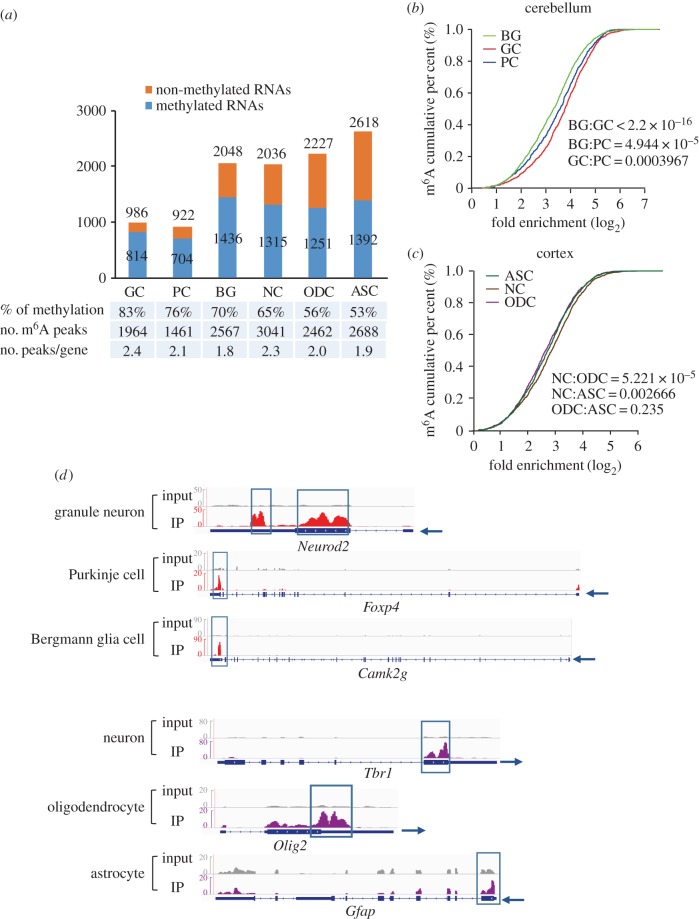
Universal RNA methylation in different types of neural cells. (*a*) Column charts showing the methylation status of different cell type-enriched genes in the cerebellum and the cortex. GC, granule neuronal cell; PC, Purkinje cell; BG, Bergmann glia cell; NC, neuronal cell; ODC, oligodendrocyte; ASC, astrocyte. The numbers of cell type-enriched genes are shown above each column; the numbers of methylated genes among those cell type-enriched genes are shown in the column. (*b,c*) Cumulative distribution function of log2-fold enrichment of m^6^A peaks in three kinds of cell type-enriched genes in mouse cerebellum (*b*) or in mouse cerebral cortex (*c*). (*d*) IGV plots showing the methylation status of representative cell type-enriched genes in the cerebellum and the cortex. The grey reads are from non-IP control (input) libraries; red and purple reads are from m^6^A-IP libraries of mouse cerebellum and cerebral cortex, respectively. Arrows show the direction of transcription. *Y*-axis represents the normalized numbers of reads count. Positions of m^6^A peaks are highlighted in the blue box.

Mellén *et al.* [68] generated cell type-enriched gene lists including 922 Purkinje cell (PC), 2084 Bergmann glial cell (BG) and 986 granule neuronal cell (GC) enriched genes. These RNAs were compared to the cerebellar m^6^A-seq dataset resulting in 814, 704 and 1436 RNAs with detectable methylation in GCs, PCs and BGs, respectively (figure 4*a*). Notably, the proportion of methylated mRNAs in GCs was much higher than that in PCs or BGs. On average, methylated mRNAs in GCs contained more m^6^A peaks than those in the other two types of cells. We also evaluated their methylation levels by assessing the fold enrichment of all methylated peaks in each type of cells. In the cerebellum, methylation levels in GCs seemed to be the highest, while BGs displayed the lowest methylation levels (figure 4*b*). In the cerebral cortex, the neuronal cells (NCs), astrocytes (ASCs) and oligodendrocytes (ODCs) were also compared with regard to m^6^A methylation levels for the cell type-enriched RNAs. We found that NC-enriched RNAs had a higher proportion of methylation, more m^6^A peaks and higher methylation levels than ASC- and ODC-enriched RNAs (figure 4*a*,*c*). In figure 4*d*, the methylation status of several representative cell type-enriched mRNAs in the cerebellum and the cortex is shown in IGV plots.

The above results further confirmed the heterogeneity of RNA methylation not only in different brain regions but also in different types of neural cells. By comparing the methylation status of cell type-enriched genes, the NCs exhibited higher methylation levels than glial cells.

### 3.5. Distinct biological functions of commonly and specifically methylated genes

Morphogenesis and functional development of the brain are accomplished through multiple gene interaction networks in a spatio-temporal-specific manner. As a crucial post-transcriptional gene regulator, the functions of m^6^A marks in mRNA should be versatile in the brain. Considering the variation of RNA methylation in various brain regions, we next conducted GO analysis to explore the biological relevance of common and specific methylation in the cerebellum and the cerebral cortex (electronic supplementary material, tables S8–S11).

**Figure 5.**
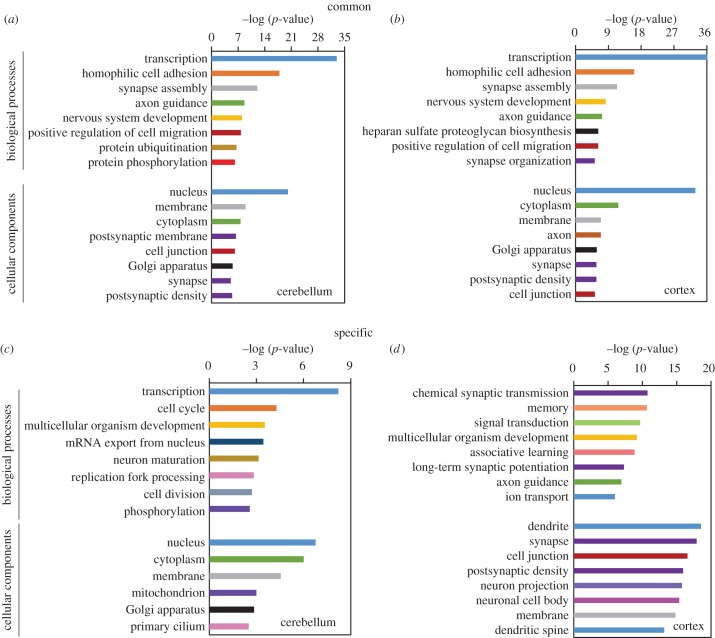
GO analysis of commonly and specifically methylated genes in mouse cerebellum and cerebral cortex. (*a,b*) GO functional analysis of the commonly methylated genes containing the top 3000 m^6^A peaks in mouse cerebellum (*a*) and cerebral cortex (*b*). (*c,d*) GO functional analysis of cerebellar (*c*) and cortical (*d*) specifically methylated genes.

The top 3000 m^6^A peaks containing CMRs were selected for GO analysis. Owing to different methylation levels of common peaks in the two brain regions, the CMRs containing the top 3000 peaks were not identical, with only half of them overlapped (electronic supplementary material, figure S7*a*). However, in mouse cerebellum and cerebral cortex, these CMRs were enriched in very similar categories including transcriptional regulation, cell adhesion, axon guidance, synapse assembly and organization, suggesting that m^6^A is an essential modification involved in diverse physiological processes (figure 5*a,b*).

In contrast to common methylation, specific methylation was involved in different functional pathways in the two brain regions. Methylated RNAs were distributed throughout various organelles mainly including nucleus, cytoplasm, Golgi apparatus and membrane. The cerebellar SMR genes, located in the nucleus, cytoplasm and membrane, were mostly involved in transcriptional regulation, cell cycle, mRNA export, neuron maturation and so on (figure 5*c*). By contrast, cortical SMR genes were mainly detected in the dendrite, synapse and cell junction where chemical synapse transmission takes place. In line with their subcellular localization, GO annotation of the cortical SMR genes included synaptic transmission, memory, learning, axon guidance and ion transport (figure 5*d*). It is worth noting that in spite of the distinct functions, the majority of the SMRs (especially cerebellar SMRs) actually had similar expression levels between the two brain regions (electronic supplementary material, figure S7*b*), which further suggested a role of m^6^A methylation in regulating spatial-specific functions in mouse brain.

### 3.6. Hyper-methylation of FMRP target mRNAs

**Figure 6.**
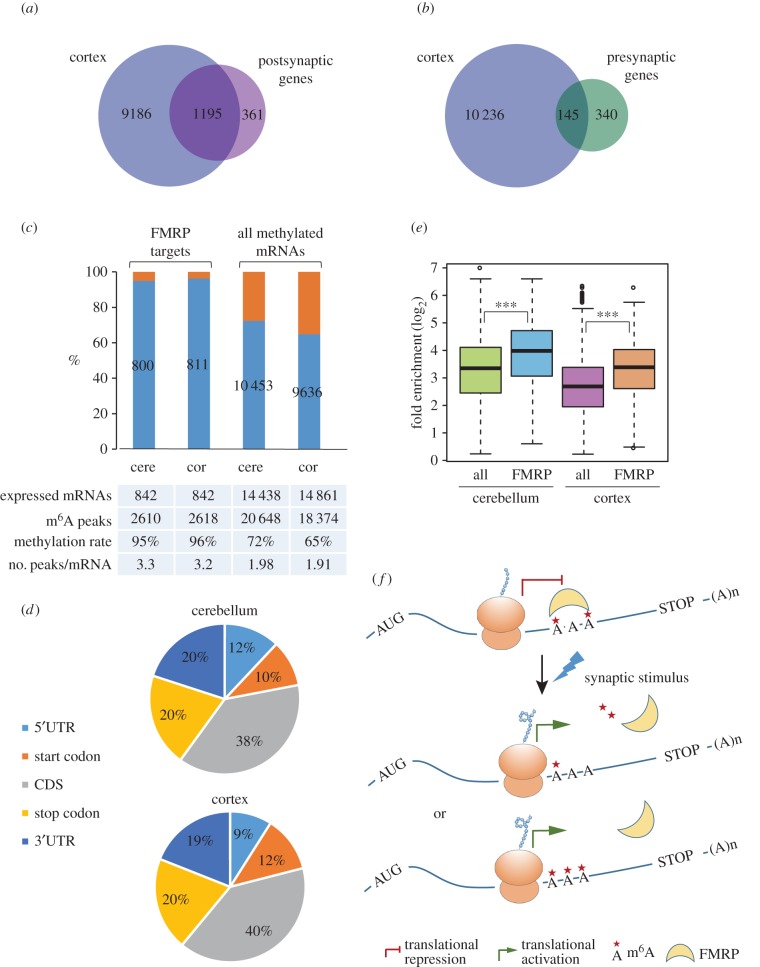
Methylation status of mouse synaptic mRNAs and FMRP target mRNAs in the mouse brain. (*a,b*) Venn diagrams showing the overlap between mouse cortical methylated genes and the genes encoding postsynaptic (*a*) or presynaptic proteins (*b*). (*c*) Column charts showing the methylation status of FMRP target mRNAs in comparison with all methylated mRNAs in mouse cerebellum and cerebral cortex. Orange bars indicate the unmethylated FMRP target mRNAs. The numbers on blue bars indicate the amount of methylated RNAs in each sample. (*d*) Pie charts showing the distribution of m^6^A peaks in methylated FMRP target mRNAs in mouse cerebellum and cerebral cortex. (*e*) Box plots showing the median methylation levels of FMRP target mRNAs in comparison with all methylated genes in mouse cerebellum and cerebral cortex, respectively. (*f*) Proposed model of the role of m^6^A RNA methylation in FMRP-induced translational repression. The target mRNAs are maintained at an appropriate RNA methylation level to be recognized by FMRP and enter a translational repression state via ribosome stalling. Upon relevant synaptic stimulus, mRNA methylation is altered, resulting from changed expression of one or more kinds of m^6^A writer, eraser or reader genes. Either increase or decrease in mRNA methylation may cause the dissociation of FMRP from target RNAs and then enable the ribosome elongation to reactivate translation. The one, two or three asterisks represent decreased, appropriate and increased methylation levels.

As revealed by GO functional annotation, CMRs and cortical SMRs were significantly enriched in the dendrite, synapse and cell junction, suggestive of the potential importance of m^6^A methylation in the synapse. We then examined the methylation status of the mRNAs encoding synaptic proteins by comparison with the mouse synaptic proteome [69,70]. As a result, 76.8% of mouse postsynaptic genes and 30% of presynaptic genes were detected with m^6^A RNA methylation (figure 6*a,b*), necessitating a requirement to explore the role of m^6^A marks in the synapse.

Local translational control is one of the key regulatory pathways for synaptic transmission; improper translational control will lead to synaptic dysfunction and subsequent cognitive disorders [71,72]. Fragile X syndrome (FXS) is such a kind of inherited disease characterized by intellectual disability and caused by *Fmr1* gene deletion or mutation, even though the mechanism is still under debate [73]. Fragile X mental retardation protein (FMRP), encoded by the *Fmr1* gene, regulates local protein translation in the synapse via its highly selective interaction with mRNAs. Various efforts have been made aiming to elucidate how FMRP recognizes its target mRNAs. Interestingly, FMRP-binding motifs on mRNAs (GGA, GAC, ACU) are highly similar to the consensus sequence of m^6^A methylation [74,75]. We then examined the possibility whether FMRP target mRNAs could be m^6^A methylated. Strikingly, among the 842 FMRP target mRNAs identified in mouse brain [76], 800 mRNAs were methylated in the cerebellum (95%) and 811 in the cortex (96%) (figure 6*c*). The numbers of m^6^A peaks in the methylated FMRP target mRNAs were much higher than the average level in all methylated mRNAs. Similar to the FMRP-binding sites in mRNAs, these peaks were mostly enriched in CDS regions, followed by the stop codon and the 3′UTR (figure 6*d*). Importantly, the median methylation levels of the methylated FMRP targets were considerably higher than that of all methylated mRNAs in both the cerebellum and the cortex (figure 6*e*). These findings implied that the m^6^A marks, especially those in the CDS regions, were most probably involved in the FMRP–RNA interactions.

## 4. Discussion

Neural development is a complex process that follows a strict spatio-temporal pattern of organization, in which both genetic and epigenetic regulators undergo tightly regulated changes [77–79]. In this study, we provided the first region-specific m^6^A RNA methylation map and explored how it correlates with the region-specific gene regulation in mouse cerebellum and cerebral cortex. Our results revealed several novel insights regarding the nature of m^6^A methylation in the brain. First, RNA methylation levels in mouse cerebellum are generally higher than those in the cerebral cortex. Second, heterogeneity of RNA methylation exists in different brain regions and different types of neural cells including the RNAs that are methylated, their methylation levels and the methylation sites. Third, common and region-specific methylation have different preferences for methylation site selection and thereby different impacts on their biological functions. Last, our results also suggested that m^6^A methylation is likely to be used for selective recognition of target mRNAs by FMRP in the synapse.

RNA methylation mainly contributes to fine-tuning of gene regulation and is sensitive to experimental conditions. Therefore, instead of cell lines, the *in vivo* model system is better to be used to address the dynamics and biological functions of RNA methylation. We have observed higher expression of methyltransferases and demethylases in mouse cerebellum than in mouse cerebral cortex. Accordingly, the cerebellum contained more methylated RNAs with higher methylation levels. Through comparative analysis between the two regions, genes were divided into those whose mRNAs were methylated in both brain regions (CMRs), and those with specific methylation either in the cerebellum or the cortex (SMRs). m^6^A methylation is a fundamental requirement for CMRs to exert their functions in various physiological processes, but specific methylation is only needed on specified occasions; therefore, the methylation levels of CMRs are significantly higher than those of SMRs. Apart from its complex anatomical structure, the brain also contains a multitude of cell types, rendering interpretation of their specific functions a challenge [80]. By comparing the methylation status of the cell type-enriched RNAs [67,68], we found that the RNA methylation level in neurons was higher than that in glia, indicating a more important role of m^6^A in neurons. Taken together, in order to precisely characterize m^6^A methylation in the brain, regional and cellular heterogeneity have to be taken into consideration. In addition, how this region specificity of m^6^A methylation is achieved awaits further investigation.

For the first time, we reported a significant enrichment of cerebellum-specific m^6^A sites at start codons, and cortex-specific m^6^A sites in the CDS. Of note, due to the heterogeneity of RNA methylation across brain regions, the region-specific features of RNA methylation may be masked if the whole brain were to be analysed altogether. Enrichment of m^6^A at start codons has been previously observed in *Arabidopsis thaliana* and rice [81–83], indicating the evolutionary conservation of such a kind of distribution. Besides m^6^A, m^1^A is another type of RNA modification enriched at start codons, which positively correlated with translation efficiency and protein expression levels [43,45]. Given the reported functions of m^6^A in translational control [10–12,84], we propose that m^6^A methylation at the start codon may be used for mRNA scanning for AUG recognition in highly metabolic active neurons [85]; however, this has to be confirmed with experimental evidence. In contrast to cerebellum-specific methylation sites, cortex-specific methylation sites are mostly detected in the CDS. Methylation analysis of FMRP target mRNAs indicates that the m^6^A marks in the CDS is probably used for selective recognition of mRNAs by FMRP.

As m^6^A modification can affect RNA structure and RNA–protein interactions, clustering of m^6^A peaks in different locations implies the versatile functions mediated via m^6^A marks. A prominent finding is that CMR genes and cortical SMR genes are enriched in the dendrites, synapses and cell junctions. Messenger RNAs localizing to these areas frequently undergo local protein translation in response to stimulus [86]. As protein translation levels correlate poorly with the mRNA abundance [87], there may exist alternative mechanisms for regulating local protein synthesis, one of which might be RNA modification. RNA m^5^C methyltransferase NSUN2 partially co-localized with FMRP, and the role of m^5^C RNA methylation in local protein translation was investigated [88]. As a result, 5.3% of postsynaptic genes, 1.9% of presynaptic genes and 10% of FMRP target mRNAs were detected with m^5^C methylation. In contrast to m^5^C, we identified that m^6^A methylation occurred at a majority of synaptic RNAs and FMRP target mRNAs with particularly high methylation levels. Moreover, in line with the fact that FMRP prefers to bind the CDS region of target mRNAs, we also observed an enrichment of the m^6^A peaks of FMRP target mRNAs in the CDS region. Strikingly, the binding motifs of FMRP protein identified from several independent studies are highly similar to the consensus sequence of m^6^A methylation, among which A appears to be the most conserved site [74,75]. Further experimental investigation, such as single-nucleotide resolution m^6^A profiling analysis and FMRP PAR-CLIP, will be necessary to determine whether FMRP is a direct m^6^A-binding protein. Based on our observation in mouse cerebellum and cerebral cortex, we deduce that m^6^A methylation is probably involved in the FMRP translation repression mechanism, and propose a dynamic model of m^6^A modification in regulating local protein translation in the synapse. As illustrated in figure 6*f*, mRNA methylation is maintained in a proper balance to be recognized by the FMRP. Through m^6^A-mediated RNA–protein interaction, FMRP represses mRNA translation via stalling ribosomal translocation. Upon physiological synaptic stimuli, one or more kinds of m^6^A-related genes may be activated and may lead to an alteration in target mRNA methylation, which probably interferes with the FMRP–RNA interaction. Finally, FMRP is released from its target mRNA and protein translation is reinitiated. It is worth noting that the mechanism of synaptic signalling in different locations might vary a lot in response to different stimuli. The m^6^A peaks in FMRP target mRNAs located in the CDS region, stop codon and 3′UTR regions may participate in synaptic signalling in different ways. Hence, more detailed *in vivo* investigation is needed to ascertain the role of m^6^A in FMRP-mediated local protein translation in a context-dependent manner.

Absence or dysfunction of *Fmr1* gene causes loss of inhibitory functions of its target RNAs and results in excessive protein synthesis, which is one of the main causes of fragile X syndrome. Accordingly, the high methylation level of FMRP target mRNAs observed in this study implies that imbalanced RNA m^6^A methylation resulting from the defect in any of the m^6^A-related genes may also be a causative factor of intellectual disorder. In support of our findings, FTO was detected in the dendrites and near-dendritic spines of the mouse brain. Li *et al.* [35] also observed impaired learning and memory in targeted FTO knockout mice. Pathophysiological stimulation, such as transient contextual fear exposure, caused a significant decrease in FTO expression and an increase in m^6^A levels of mRNA in synapses [34]. Furthermore, *in situ* FTO depletion or FTO knock-down resulted in increased m^6^A methylation and enhanced memory formation in both mouse hippocampus and prefrontal cortex [34,89]. Recently, an SNP in the *ALKBH5* gene was identified in association with major mental disorders in the Chinese Han population [90]. *FTO* genetic variants were also reported associated with a high risk of Alzheimer's disease and impaired brain functions [91,92]. Therefore, it is of paramount importance to characterize m^6^A RNA methylation in greater detail, with much attention to its spatio-temporal specificity and cellular heterogeneity in the brain.

Nevertheless, it should be noted that, despite the several novel insights obtained, there are several limitations in this study. First, the common and specific methylation described above does not reflect the situation of the whole mouse brain because only the cerebellum and the cerebral cortex were included for comparison. Second, the m^6^A-seq analysis used in this study cannot tell the exact location of methylation sites at base level, therefore m^6^A analysis of single-nucleotide resolution will be necessary in order to precisely evaluate the role of m^6^A at specified methylation sites. Third, the RNA methylation in various types of cells as evaluated only with the cell type-enriched genes, thus the information of the low-abundance but highly methylated RNAs was missing. In addition, because we used a mixture of multiple types of cells for m^6^A-seq, the methylation status of each gene in different cells cannot be characterized here. Therefore, to thoroughly study the heterogeneity of RNA methylation and related functions, there is a pressing need to develop an accurate, sensitive and quantitative method for m^6^A analysis.

## 5. Conclusion

In summary, we analysed the region-specific methylation profiles and characterized their functions in mouse cerebellum and cerebral cortex. Our results imply that RNA methylation exhibits different characteristics across different brain regions or different types of neural cells. As a representative epitranscriptomic mark, m^6^A is a newly identified element in the region-specific gene regulatory network in the mouse brain. Elucidation of an m^6^A-dependent regulatory network in the brain should greatly facilitate our understanding of brain development and help unravel the aetiology of neurological diseases, which in turn might be able to offer novel diagnostic or therapeutic targets in the future.

## Supplementary Material

Supplementary material Chang

## Supplementary Material

Supplementary tables Chang

## Data accessibility

The datasets supporting the conclusions of this article are available in the Genome Sequence Archive (GSA) of BIG Data Center, Beijing Institute of Genomics (BIG), Chinese Academy of Sciences and are publicly accessible at http://gsa.big.ac.cn (Accession no. PRJCA000272).
