# Supplementary material for: Region-specific RNA m6A methylation represents a new layer of control in the gene regulatory network in the mouse brain
Source: Open Biol. 2017 Sep 20;7(9):170166. doi: 10.1098/rsob.170166 (PMC5627058; doi:10.1098/rsob.170166)
Supplement: Supplementary material Chang [file rsob170166supp1.pdf]

SUPPLEMENTARY MATERIALS

Mengqi Chang[1]*, Hongyi Lv[2,3]*, Weilong Zhang[4*], Chunhui Ma[1], Xue He[1], Shunli Zhao[1], Zhi-Wei Zhang [1], Yi-xin Zeng[4], Shuhui Song[2], Yamei Niu[1#], Wei-Min Tong[1#] (2017) Region-Specific RNA m^{6}A Methylation Represents a New Layer of Control in the Gene Regulatory Network in the Mouse Brain. Open Biology 10.1098/rsob.170166

SUPPLEMENTARY DATA INCLUDES:

Supplementary figures and legends, supplementary table S1-S3, and supplementary table S4-S12 (see attached excel file).

SUPPLEMENTARY FIGURES

a

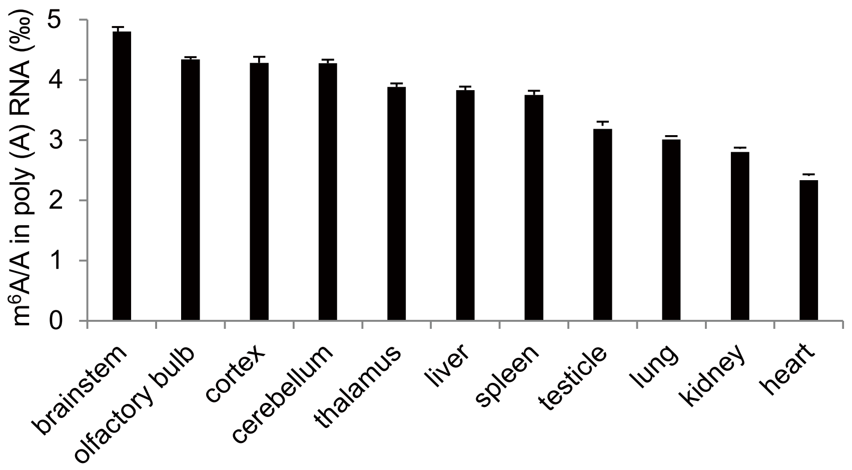

b

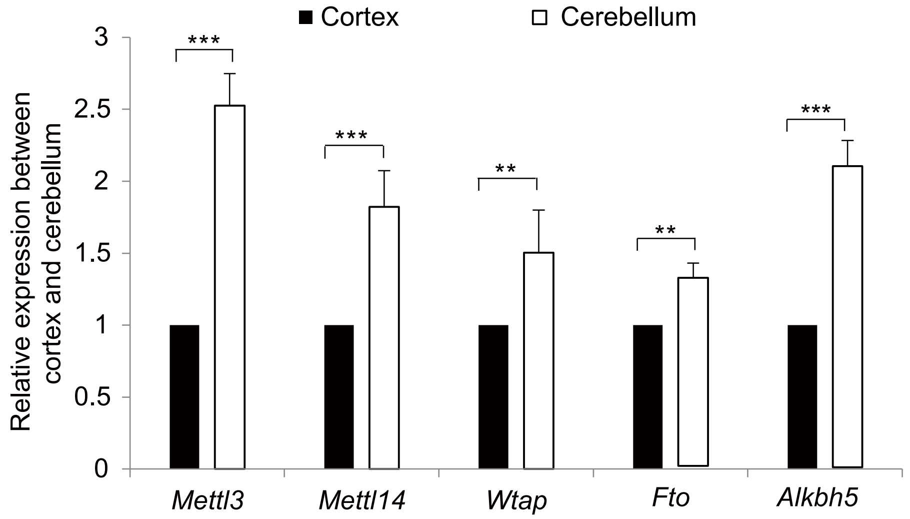

Figure S1, related to Figure 1. RNA methylation profiles across mouse tissues and RNA expression profiles of m^{6}A writers and erasers. (a) UHPLC-MS/MS analysis of poly(A) RNA in adult mouse tissues for m^{6}A /A ratio. The experiments were repeated for three times using 10 male mice in total. Representative data was shown here as average values + SEM. (b) Real-time PCR analysis to compare the RNA expression of m^{6}A writer and eraser genes in mouse cerebellum and cerebral cortex. The ratio of RNA expression between cerebellum and cortex was shown here as mean value + SEM from 3 male and 3 female 3-month-old mice. **, p value<0.01, ***, p value<0.001.

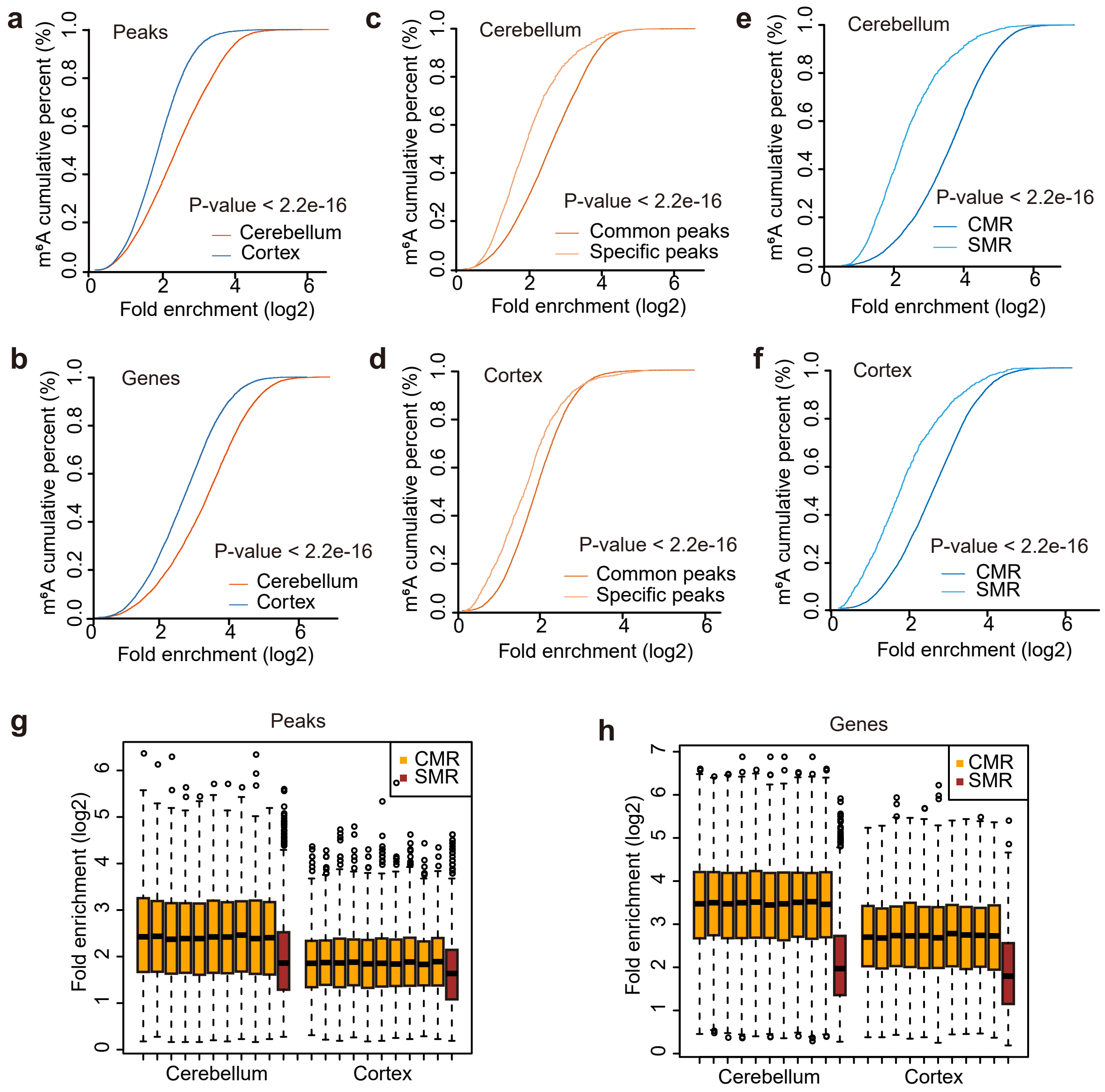

Figure S2, related to Figure 2. Cumulative curves comparing the methylation levels between cerebellum and cortex, or common and specific methylation. (a, b) Cumulative distribution function of log2-fold enrichment of m^{6}A peaks to compare the methylation levels between cerebellar and cortical RNAs at peaks level (a) and genes level (b). (c, d) Cumulative distribution function of log2-fold enrichment of common and specific m^{6}A peaks in mouse cerebellum (c) and cerebral cortex (d). (e, f) Cumulative distribution function of log2-fold enrichment of m^{6}A peaks to compare the methylation levels between CMRs and SMRs in mouse cerebellum (e) and cerebral cortex (f). CMR, commonly methylated RNA. SMR, specifically methylated RNA. Wilcoxon test was performed for statistical analysis. (g, h) Box plots showing the methylation levels of CMRs and SMRs by comparing the fold enrichment at peaks level (g) or genes level (h). The same number of peaks or genes as those of SMRs were randomly extracted from CMRs, and compared with SMRs for 10 times in total.

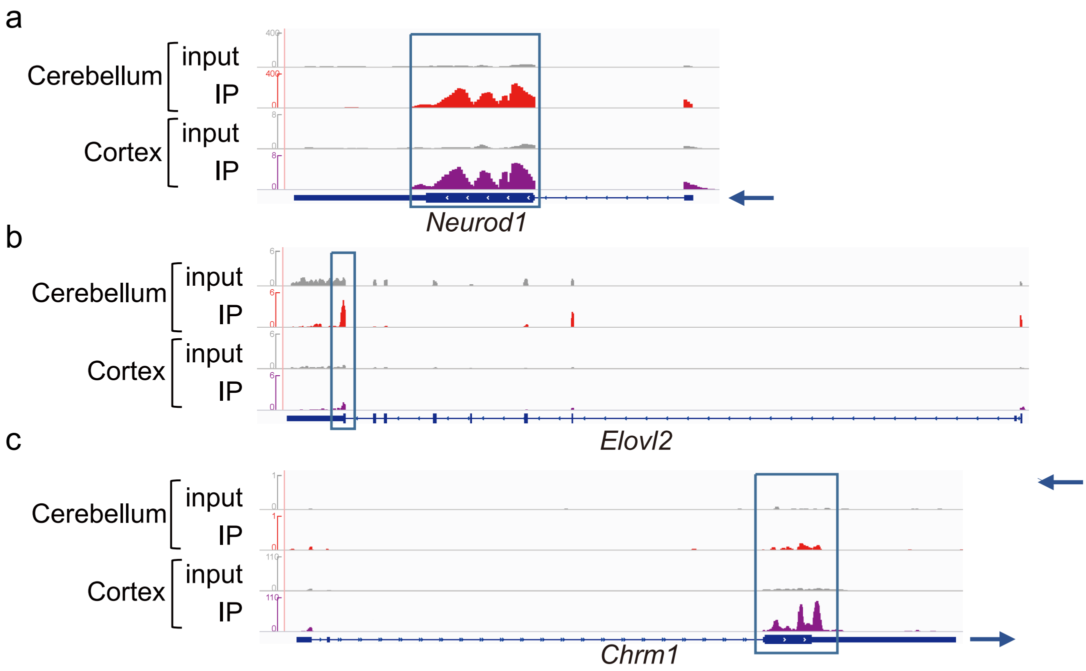

Figure S3, related to Figure 2. IGV plots showing examples of common (a), cerebellar (b) and cortical specifically (c) methylated transcripts. The grey reads are from non-IP control (input) libraries; red and purple reads are from m⁶A-IP libraries of mouse cerebellum and cerebral cortex, respectively. Arrows show the direction of transcription. Y-axis represents normalized numbers of reads count. Positions of m⁶A peaks are highlighted in blue box.

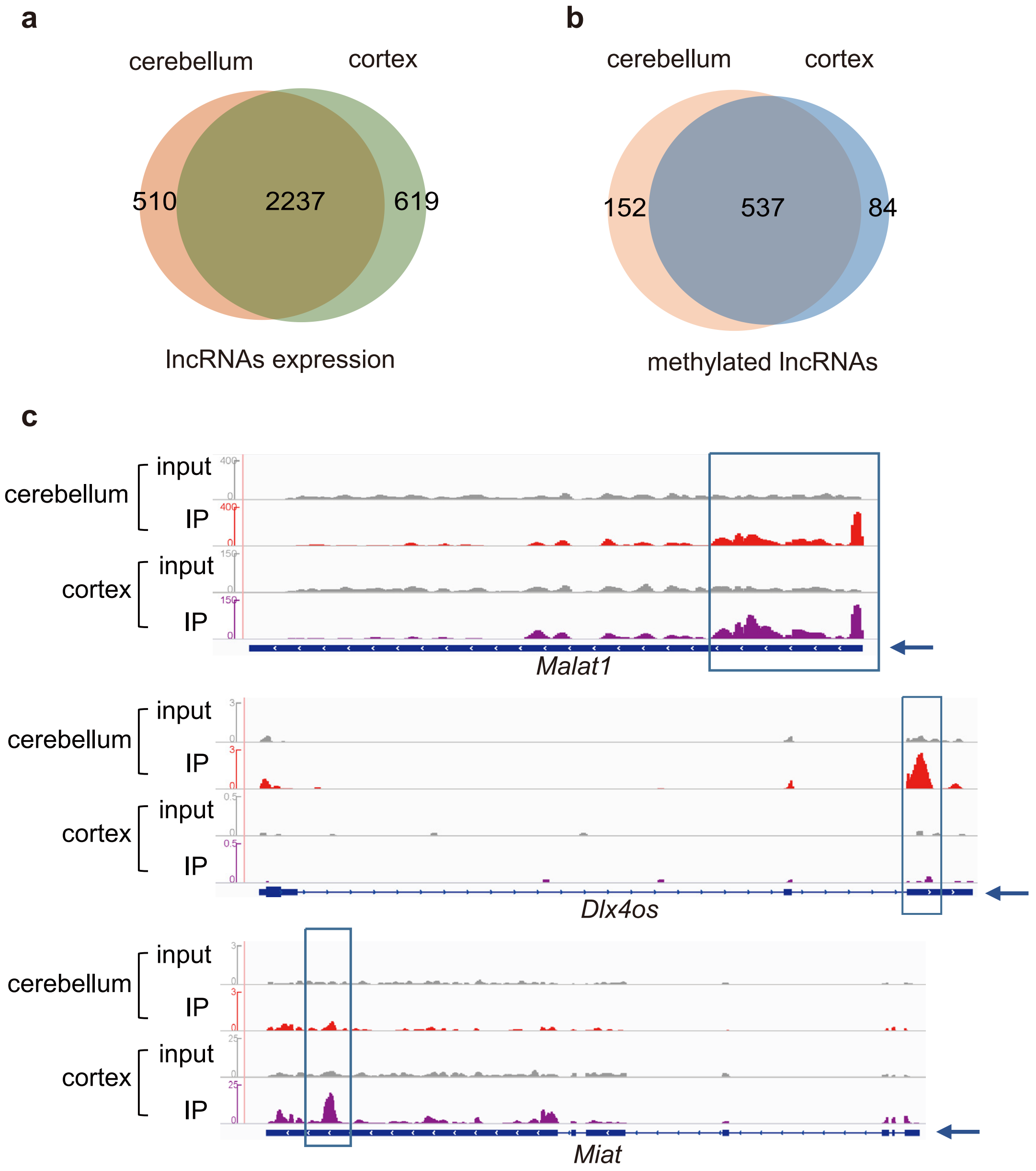

Figure S4, related to Figure 2. Comparative analysis of m⁶A methylated lncRNAs between mouse cerebellum and cerebral cortex. (a) Venn diagram showing the numbers of lncRNAs commonly or specifically expressed in mouse cerebellum and cerebral cortex. (b) Venn diagram showing the numbers of commonly or specifically methylated lncRNAs between cerebellum and cerebral cortex. (c) IGV plots showing examples of commonly (upper panel), cerebellar (middle panel) and cortical specifically (lower panel) methylated lncRNAs. The grey reads are from non-IP control (input) libraries; red and purple reads are from m⁶A-IP libraries of mouse cerebellum and cerebral cortex, respectively. Arrows show the direction of transcription. Y-axis represents normalized numbers of reads count. Positions of m⁶A peaks are highlighted in blue box.

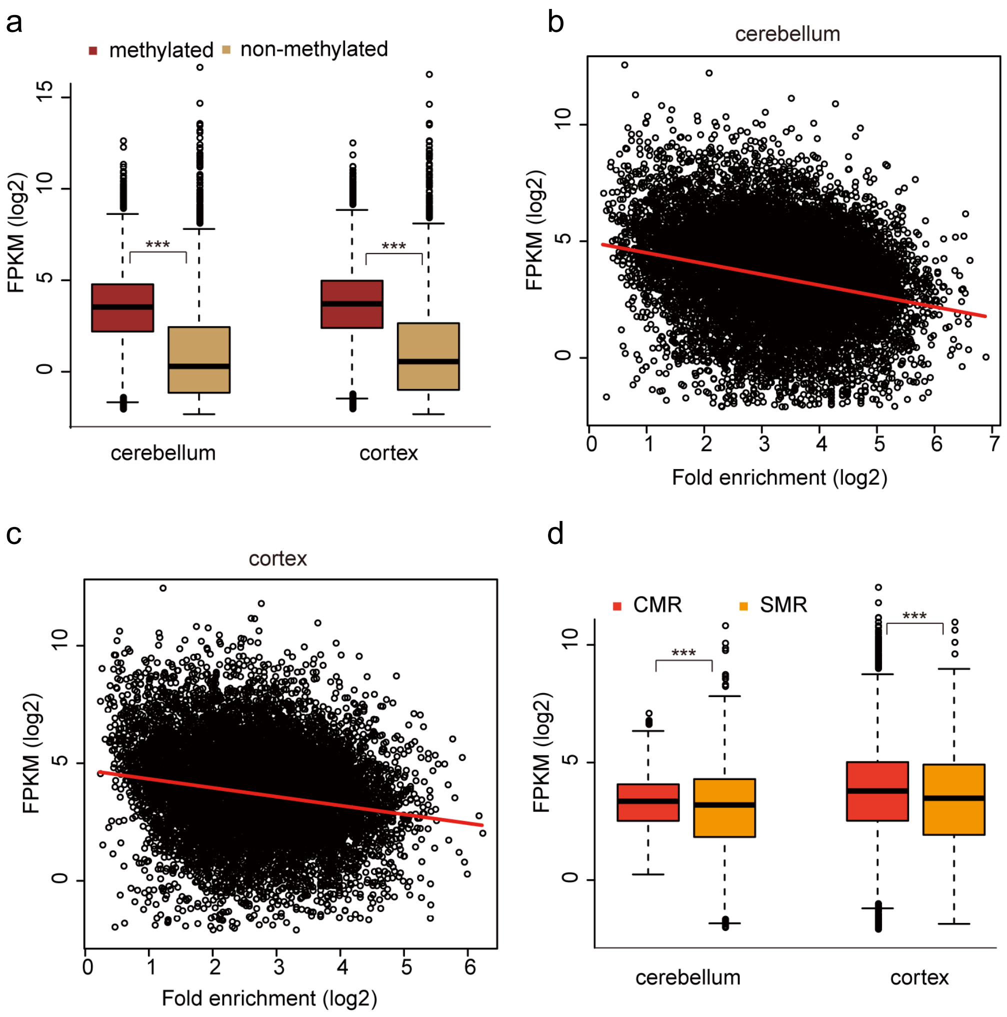

Figure S5, related to Figure 2. Correlation analysis between RNA methylation and expression. (a) Box plots showing the median expression levels of methylated genes and non-methylated genes in mouse cerebellum and cerebral cortex. (b, c) Scatter diagrams showing correlation between m^{6}A peak enrichment (fold enrichment log2) and RNA abundance (FPKM log2) in mouse cerebellum (b) and cerebral cortex (c). (d) Box plot showing the relative expression levels of CMRs and SMRs in mouse cerebellum and cerebral cortex. CMR, commonly methylated RNA. SMR, specifically methylated RNA. *** indicates p value <2.2e-16. Wilcoxon test was performed for statistical analysis.

a

Cerebellum			
motifs	P-value	% of targets	% of background
GGACU	1e-383	50.50%	35.45%
AUCGG	1e-117	45.42%	37.13%
UGGAG	1e-78	53.24%	46.35%
CGGUG	1e-77	28.92%	23.00%
UAUGA	1e-74	22.93%	17.62%

b

Cortex			
motifs	P-value	% of targets	% of background
UGGAC	1e-226	37.89%	25.95%
CGACG	1e-102	35.32%	27.29%
CAGCG	1e-72	35.90%	29.09%
GAUGA	1e-67	39.11%	32.37%
UACCG	1e-46	17.84%	13.67%

c

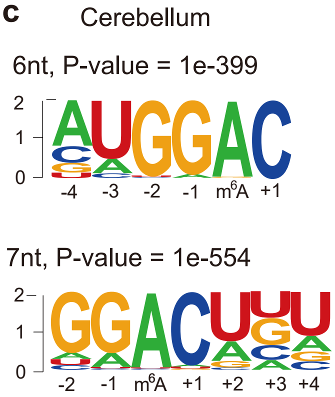

d

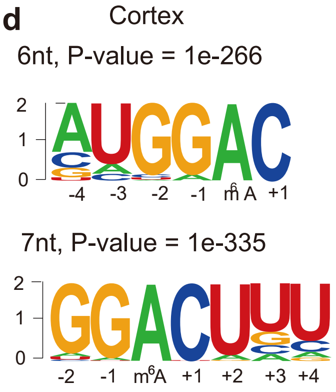

e

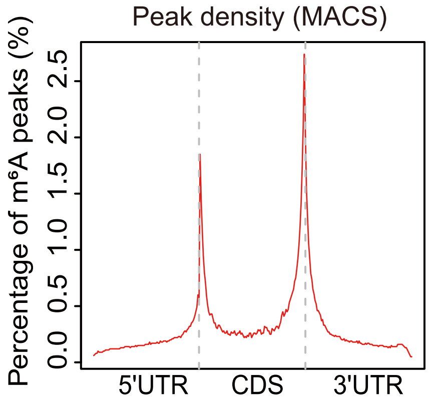

f

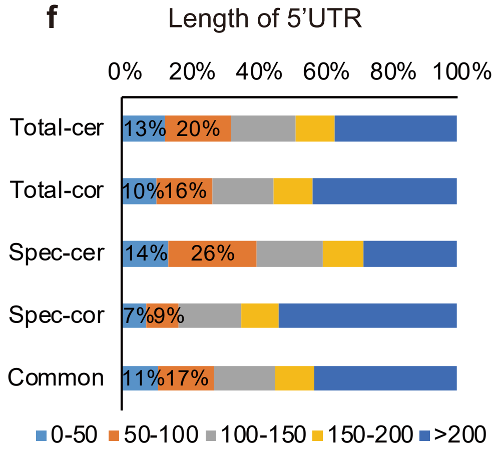

Figure S6, related to Figure 3. Consensus sequence and distribution of m⁶A peaks. (a, b) Lists of the top 5 consensus sequence (5nt) identified in m⁶A peaks in mouse cerebellum (a) and cortex (b). (c, d) Consensus sequence (upper panel, 6nt) and (lower panel, 7nt) identified in m⁶A peaks in mouse cerebellum (c) and cortex (d). (e) Distribution of cerebellar m⁶A peaks density along the transcripts as analyzed using MACS software. (f) Statistics of 5'UTR length of the transcripts including m⁶A methylation sites surrounding start codons.

a

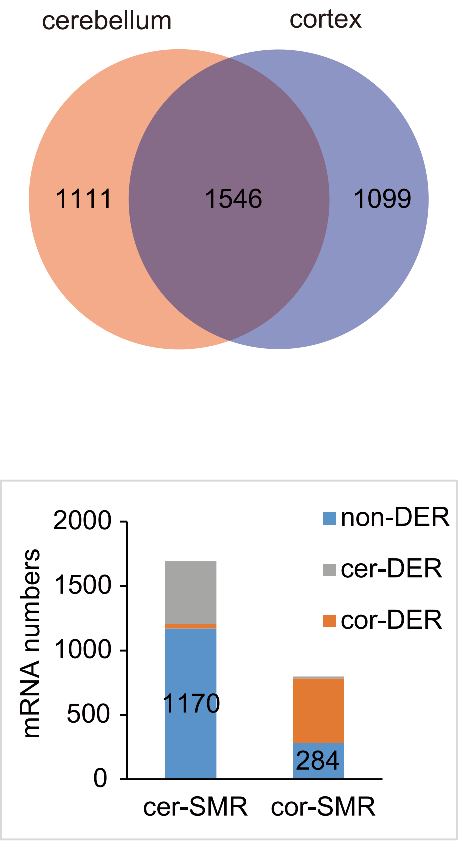

Figure S7, related to Figure 5. Characterization of commonly and specifically methylated genes in mouse cerebellum and cerebral cortex. (a) Venn diagram showing the overlap between the top 3000 m^{6}A peaks-containing, commonly methylated genes in cerebellum and cerebral cortex. (b) Status of differential expression of specifically methylated genes in cerebellum and cortex. cer-SMR, specifically methylated RNAs in cerebellum; cor-SMR, specifically methylated RNAs in cerebral cortex. non-DER, non-differentially expressed RNAs. cor-DER, differentially expressed RNAs with higher expression in cortex; cer-DER, differentially expressed RNAs with higher expression in cerebellum. $p < 0.05$ was set to judge the significance of differential expression.

SUPPLEMENTARY TABLES

Table S1. Information of m^{6}A-seq data quality and processing.

	cerebellum_1		cerebellum_2		cortex_1		cortex_2	
	INPUT	IP	INPUT	IP	INPUT	IP	INPUT	IP
Raw_data_reads	81898124	59903174	46652031	62481982	65790997	62320609	57505666	56331522
Clean reads	74423860	53808924	42799584	57320663	59574371	56302825	52149919	50709485
Ratio	91%	90%	92%	92%	91%	90%	91%	90%
length > 50bp	70019400	50642244	40539120	54549366	56093974	53102625	49029708	47889156
Ratio	94%	94%	95%	95%	94%	94%	94%	94%
#mapped_reads	61013860	45966387	35225594	47868528	48727738	47488219	40713068	40681156
Ratio	87%	91%	87%	88%	87%	89%	83%	85%
#unique_mapped reads	58223489	44619904	33691142	46323848	46625042	45981248	38713635	39515641
Ratio	83%	88%	83%	85%	83%	87%	79%	83%

Table S2. Statistics of RNA-seq data of mouse cerebellum and cerebral cortex.

	Total		Specific		Common
samples	cerebellum	cortex	cerebellum	cortex	Cerebellum/cortex
Genes	19657	19760	1656	1759	18001
mRNA	14438	14861	426	849	14012
lncRNA	2856	2747	619	510	2237
others	2363	2152	611	400	1752

Table S3. Statistics of methylated peaks and genes in mouse cerebellum and cerebral cortex as analyzed from the two biological repeats of m^{6}A-seq.

	Total		Region-specific		Common	
samples	cerebellum	cortex	cerebellum	cortex	cerebellum	cortex
genes	11324	10381	1728	805	9519	9519
peaks	21814	19746	2018	1043	16576	16989
peaks/gene	1.93	1.90	1.17	1.30	1.74	1.78
mRNA	10453	9636	1559	712	8924	8924
% methylation	72.4%	64.8%	ND	ND	ND	ND
peaks	20648	18734	1826	932	15806	16209
peaks/mRNA	1.98	1.94	1.17	1.31	1.77	1.82
lncRNA	777	664	152	84	537	537
% methylation	27.2%	24.2%	ND	ND	ND	ND
Peaks	1045	916	175	101	703	712
peaks/lncRNA	1.34	1.38	1.15	1.20	1.31	1.33

(ND: no data)

Supplemental Table S4-S12 are attached as excel files.

Table S4. List of commonly methylated m^{6}Apeaks in mouse cerebellum.

Table S5. List of commonly methylated m^{6}A peaks in mouse cerebral cortex.

Table S6. List of specifically methylated m^{6}A peaks in mouse cerebellum.

Table S7. List of specifically methylated m^{6}A peaks in mouse cerebral cortex.

Table S8. GO analysis of commonly methylated genes in mouse cerebellum.

Table S9. GO analysis of commonly methylated genes in mouse cerebral cortex.

Table S10. GO analysis of specifically methylated genes in mouse cerebellum.

Table S11. GO analysis of specifically methylated genes in mouse cerebral cortex.

Table S12. Sequence of primers used for RT-qPCR.
